# The relationship between serum C-reactive protein and senile hypertension

**DOI:** 10.1186/s12872-022-02948-4

**Published:** 2022-11-24

**Authors:** Le He, Chenyang Fan, Gang Li

**Affiliations:** 1 North China University of Science and Technology, Tangshan, Hebei China; 2 Hebei North University, Zhangjiakou, Hebei China; 3 Division of Cardiology, Institute of Geriatric Diseases, Hebei General Hospital, Shijiazhuang, Hebei China

**Keywords:** C-reactive protein, Hypertension, Elderly, Inflammation

## Abstract

**Background:**

Hypertension has become an important health risk factor in the twenty-first century, especially for the elderly. Studies have confirmed that inflammation is involved in the development of hypertension and that the inflammatory marker C-reactive protein(CRP) is significantly associated with hypertension. Therefore, in this study, we aimed to explore the CRP correlation with hypertension in the elderly.

**Methods:**

Serum CRP levels were measured in 196 hospitalized patients, and the CRP level was used as a criterion to divide them into the group with elevated CRP (> 10 mmol/L, *n* = 120) and the group with normal CRP (0 < CRP ≤ 10 mmol/L, *n* = 76). and the patient's baseline characteristics were collected and compared between the groups, and the correlation between CRP and other factors and hypertension in the elderly was analyzed by multi-factor logistic regression.

**Results:**

The prevalence of hypertension, coronary artery disease and joint reactive inflammation was significantly higher in the group with elevated CRP. There was also a significant association between the use of alcohol, low density lipoprotein cholesterol (LDL-C) and steroids and elevated CRP; logistic regression showed that elevated CRP (OR = 2.13, 95% CI: 1.14–3.99, *p* = 0.019), body mass index (BMI) (OR = 1.10, 95% CI:1.01–1.90, *p* = 0.030), diabetes (OR = 2.68, 95% CI:1.24–5.79, *p* = 0.012) were positively associated with hypertension, while statins use was negatively associated with hypertension (OR = 0.49, 95% CI: 0.25–0.94, *p* = 0.013).

**Conclusions:**

Elevated CRP, BMI, and diabetes are positively associated with hypertension in the elderly, and early screening for CRP and initiation of treatment may help prevent further inflammatory responses in hypertension.

## Highlights

1. Inflammation plays an important role in vascular remodeling and myocardial remodeling in hypertension. Our study found that CRP, as a marker of systemic inflammation, was significantly positively correlated with hypertension in the elderly.

2. The most common phenomenon in elderly patients is co-morbidity. we found that the proportion of hypertension, coronary heart disease and arthritis in patients with elevated CRP was significantly higher than that in patients with normal CRP.

3. Inflammation is related to hypertension, and CRP is one of the inflammatory markers. Early screening and intervention of CRP may delay the progression of hypertension.

## Introduction

The incidence and mortality of cardiovascular and cerebrovascular diseases are.

increasing year by year. According to statistics, China has now become the country with the highest cardiovascular mortality [1]. Hypertension is an important risk factor for cardiovascular and cerebrovascular diseases. Due to the continuous progress of population aging, the prevalence of hypertension in the elderly over 65 years old is increasing, while the prevalence of hypertension in the elderly population over 80 years old is more than 90% [2, 3]. Therefore, hypertension has been an urgent problem to solve and prevent. Hypertension is considered to be an inflammatory disease [4, 5]. Inflammatory response plays an important role in vascular remodeling and myocardial remodeling in hypertension. In 2001, Bautista et al. [6] found that CRP levels remained strongly associated with hypertension and first clearly proposed CRP concentrations as an independent risk factor for hypertension, but this finding was numerous questioned due to the small sample size of their study and the fact that the prevalence of hypertension was as high as 46%, higher than the prevalence at that time. Subsequently, Sesso et al. [7], after following 20,525 normotensive women for an average of 7–8 years, observed a total of 5365 hypertensive patients, and baseline CRP concentrations were higher in these populations than in non-hypertensive populations, and even after adjusting for multiple risk factors, baseline CRP was still independently associated with hypertensive events, thus proposing CRP as an independent predictor of hypertension [8, 9]. Subsequently, scholars from home and abroad conducted cross-sectional and prospective studies on the association between CRP and hypertension, all of which confirmed that CRP was independently associated with hypertension and that CRP was one of the predictors of hypertension. However, most of the study subjects included in their experiment were young and middle-aged men and women, so the experimental results suggest that CRP is independently associated with hypertension in younger age groups. However, fewer experiments have been conducted nationally and internationally to study the correlation between CRP levels and hypertension in older adults over 65 years of age, especially in the elderly. Therefore, the main purpose of our study was to explore the relationship between CRP and hypertension in the elderly, and to provide support for the prevention of early hypertension and common diseases in the elderly.

## Methodology

### Study Sample

**Fig. 1:**
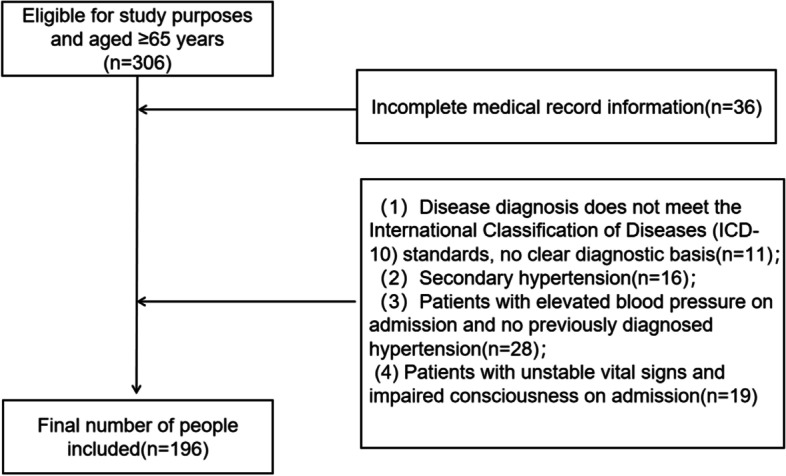
Population flow chart of the correlation study between serum liver enzymes and hypertension

We selected 306 elderly (age ≥ 65 years) patients who visited the Hebei General Hospital between November 2020 and November 2021, and 196 cases with complete data and meeting the inclusion and exclusion criteria as the study population (Fig. 1). All study subjects were excluded from the use of anti-hypertensive drugs. The normal range of CRP in our hospital laboratory was 0 < CRP ≤ 10 mmol/L. Based on this criterion, we divided the study subjects into a group with elevated CRP (CRP > 10 mmol/L, *N* = 120) and a group with normal CRP (0 < CRP ≤ 10 mmol/L, *N* = 76). Diagnostic criteria for geriatric hypertension were performed according to the *Chinese Guidelines for the Management of Geriatric Hypertension 2019* [3], That is, in elderly people aged ≥ 65 years, SBP ≥ 140 mmHg (1 mmHg = 0.133 kPa) and/or DBP ≥ 90 mmHg measured three times on different days without antihypertensive drugs can be diagnosed as geriatric hypertension. Older adults who have been diagnosed with hypertension and are receiving antihypertensive medication, although their blood pressure is < 140/90 mmHg, should be diagnosed with geriatric hypertension. In addition, All study subjects signed informed consent, and this study was approved by our hospital ethics committee.

### Sample collection and analysis

We collected general information about the study subjects, including age, sex, height, weight, and calculated the corresponding BMI. We mainly collected the following data from the study subjects: (1) basic information: age, sex, height, weight, and calculated the corresponding BMI; (2) systolic and diastolic blood pressure; (3) personal history: history of smoking and alcohol consumption; (4) related diseases: hypertension, diabetes mellitus, hyperlipidemia, coronary heart disease, cerebrovascular disease, carotid artery stenosis, peripheral arterial disease, hypothyroidism, congestive heart failure, arthritis, other rheumatologic disorders, asthma, Liver disease.(4) History of medications, such as aspirin, non-steroidal anti-inflammatory drugs(NSAIDs), statins, steroids and immunosuppressive drugs; (5) Relevant laboratory tests: all study subjects fasted for 8–12 h before the morning collection of elbow venous blood, and the main collection included CRP, white blood cell count (WBC), total cholesterol (TC), LDL-C, high density lipoprotein cholesterol (HDL-C) and triglyceride (TG), etc. Immuno-dispersive turbidimetry was used to determine CRP.

### Statistical methods

Statistical analysis was completed using IBM SPSS 26.0 software. For the 196 subjects included in this study, the statistics of normally distributed measures were expressed as mean ± standard deviation and analyzed by the independent samples t-test, while the non-normal distribution was expressed as median (interquartile spacing) and analyzed by the Mann–Whitney U test; the count data were expressed as percentages and the chi-square test was used for comparison between groups; To exclude the effect of confounding factors, we used multifactorial logistic regression to analyze the relationship between normal and elevated CRP, age, sex, BMI, statins, diabetes mellitus, chronic obstructive pulmonary disease, and asthma and hypertension in the elderly.

## Results

### Baseline characteristics of the study cohort

A total of 196 elderly patients were included in this study, 120 (61.2%) in the group with elevated CRP, of which 64 (53.3%) were men and 56 (46.7%) were women, aged 65–92 years, and 76 (38.8%) in the group with normal CRP, of which 45 (59.2%) were men and 31 (40.8%) were women, aged 65–94 years old. There were differences between the two groups in SBP, DBP and history of alcohol consumption, and the group with elevated CRP was larger than the normal group, with statistically significant differences (*P* < 0.05), while there were no significant differences between age, gender, BMI and history of smoking, with no statistically significant differences (*P* > 0.05).For laboratory indices, LDL-C levels were higher in the group with elevated CRP than in the normal group (*P* < 0.05), and there was no statistically significant difference between the two groups in TC, TG, HDL-C, and WBC (*P* > 0.05) (Table 1).

### Comparison of drug use history

In terms of medication history, there was a difference between the two groups in taking steroid hormone drugs, with the elevated group (45.0%) being greater than the normal group (30.3%), with a statistically significant difference (*P* < 0.05); in taking aspirin, statins, non-steroidal anti-inflammatory drugs, and immunosuppressants, the difference was not statistically significant. (*P* > 0.05) (Table 2).

### Comparison of co-morbidities

Analysis of co-morbidities showed that we found significant differences between the two groups suffering from hypertension, coronary heart disease, and arthritis, and the group with elevated CRP was larger than the normal group, with statistically significant differences (*P* < 0.05); between the two groups with diabetes, hyperlipidemia, cerebrovascular disease, carotid stenosis, peripheral arterial disease, hypothyroidism, congestive heart failure, other rheumatic diseases, psychosomatic diseases, chronic obstructive pulmonary disease, asthma, and liver disease, the differences were not statistically significant (*P* > 0.05) (Table 3).

To exclude the effect of confounding factors, we used multifactorial logistic regression to analyze the relationship between CRP and other related factors and hypertension, and the results showed that BMI (OR = 1.01,95%CI:1.01–1.19), elevated CRP (OR = 2.13,95%CI:1.14–3.99), diabetes mellitus (OR = 2.68,95% CI:1.24–5.79) were positively associated with hypertension (*P* < 0.05), statin use was a protective factor for hypertension (OR = 0.49, 95% CI: 0.25–0.94, *P* < 0.05), and age, gender, Chronic obstructive pulmonary disease and asthma were not significantly associated with hypertension (*P* > 0. 05) (Table 4).

## Discussion

CRP as a sensitive inflammatory marker is involved in the non-specific inflammatory response of the body. Elevated CRP not only predicts a much higher probability of developing hypertension from normal blood pressure or prehypertension, but in patients with diagnosed hypertension, elevated CRP often indicates poor blood pressure control or the development of hypertensive complications, and in patients with high baseline blood pressure, both systolic and diastolic, CRP is mostly elevated [10]. Several cross-sectional and cohort studies have reported an association between CRP and hypertension, and their studies all support the association between CRP and hypertension, and CRP may serve as a predictor of hypertension [7, 9, 11–13]. Savoia et al. [14] found that high levels of CRP upregulate angiotensin receptor expression, promote plasminogen activator inhibitor-1 (PAI-1) production, and activate vascular smooth muscle, promote the release of inflammatory factors, attenuate the response of vascular endothelial cells to diastolic substances, reduce nitric oxide production, and increase vascular resistance. In our study, we found that in the elderly population, blood pressure levels were significantly higher in the group with elevated CRP than in the normal group, and the prevalence of hypertension was higher in the group with elevated CRP than in the normal group, with statistically significant differences (*P* < 0.05), and logistic regression also showed an independent positive association between elevated CRP and the risk of hypertension (OR = 2.13, 95% CI: 1.14–3.99, *P* < 0.05). In a study of the effect on the risk of hypertension in the US retired population, which included 2924 participants, logistic regression showed that CRP could be used as a predictor of hypertension in women [15]. In another study of CRP and hypertension in Japanese men [16], 2991 male workers without hypertension were evaluated, and the risk ratio (HR) for the occurrence of hypertensive events was estimated based on the quartiles of CRP, and 579 (19.4%) subjects developed hypertension during the 5-year follow-up period, and the incidence of hypertension increased with increasing levels of CRP, and the HR increased significantly with increasing CRP levels. This may imply that the development of hypertension involves a pathological process in which inflammation is involved and that the inflammatory response may be the direct cause of hypertension.

As we age, our physiological functions gradually decline. In addition to hypertension, the coexistence of various diseases such as coronary heart disease and diabetes is becoming more and more evident in the elderly population. According to a survey conducted by the Journal of the American Medical Family Council in 2013, about 2/3 of American senior citizens have co-morbidities [17]. A large body of evidence suggests that CRP is closely associated with dyslipidemia, diabetes, cardiovascular disease, and metabolic syndrome, and can be used as an indicator for early screening or long-term monitoring to provide some basis for patient prognosis [18–20]. CRP acts as a marker of inflammation and numerous studies have shown that CRP is significantly associated with cardiovascular disease. When an inflammatory response is generated, CRP is induced by interleukin 1 (IL-1), interleukin-6 (IL-6) and tumor necrosis factor (T NF-α), mainly by the liver. CRP is often elevated in nonalcoholic fatty liver disease, suggesting that the accumulation of hepatic adipose tissue is associated with enhanced inflammation-mediated oxidative stress. CRP induces a significant increase in the expression of intercellular adhesion molecule-1 (ICAM-1) and vascular cell adhesion molecule-1 (VCAM-1), which accelerates the inflammatory response in atherosclerosis. At the same time, high concentrations of CRP can promote thickening of the intima and atherosclerosis, leading to remodeling of hypertensive vessels [21, 22]. In addition, CRP stimulates endothelial cells, megalophils and polymorphonuclear cells to secrete endothelin-1 (ET-1), interleukin-6 (IL-6) and vasoconstrictor peptide, resulting in vasoconstriction [22]. Coronary artery disease was significantly associated with elevated CRP levels in this study, and serum LDL-C was also associated with elevated CRP, with statistically significant differences (*P* < 0.05). This is consistent with previous research[23]. In addition, CRP has been shown to be an independent predictor of the risk of myocardial infarction, stroke and peripheral vascular disease and can be used to predict future risk in patients with stable and unstable angina [24]. In addition, we found that the prevalence of diabetes was higher in the group with elevated CRP than in the normal group, and although it was not statistically significant, diabetes was positively associated with the development of hypertension (OR = 2.68,95%CI:1.24–5.79, *P* < 0.05). Studies have concluded that CRP concentrations tend to be higher in patients with type 2 diabetes and that CRP can disrupt insulin homeostasis and increase the risk of disease by blocking the major cascade of downstream insulin signaling [25], and in vitro studies have also shown an association between serum CRP levels and β-cell dysfunction and insulin resistance[26]. In addition, the development of arthritis is undoubtedly an important cause of reduced quality of life in elderly or even elderly patients, and arthritis as an inflammatory condition is closely associated with CRP. For example, patients with rheumatoid arthritis usually have persistently elevated CRP levels, with baseline CRP levels often exceeding 20 mg/L in randomized clinical trials of drugs for rheumatoid arthritis [16], however, retrospective and observational studies have found that many patients with rheumatoid arthritis have normal CRP levels despite exhibiting disease activity, suggesting that CRP levels only reflect signs of disease activity [27, 28].

Obesity as a risk factor for hypertension has become a globally recognized fact, and in this study, BMI was higher in the group with elevated CRP than in the normal group, and although the difference was not statistically significant (*P* > 0.05), studies have shown that pro-inflammatory proteins such as interleukin 6 (IL-6), which is broken down by adipose tissue in obese individuals, can increase CRP levels in the liver [28]. In 2003, Hotamisligil [29] proposed that obesity is a state of systemic chronic low-grade inflammation induced by different inflammatory factors. CRP, as a relatively sensitive marker of chronic inflammation, reflects the activity and amount of inflammatory factors, and is involved in the pathogenesis of obesity by regulating lipid metabolism, increasing the inflammatory response, decreasing tissue sensitivity to insulin, stimulating insulin secretion, and promoting lipid synthesis, further aggravating obesity [30]. We also found that steroid use was proportionally higher in the group with elevated CRP (*p* < 0.05). As an inhibitor of hydroxy-methyl-glutaryl coenzyme A reductase, statin not only reduces LDL-C levels, but also decreases inflammation, reduces oxidative stress, and has a protective effect on the cardiovascular system. For example, Rosuvastatin has been shown to be effective not only in improving lipids, but also in lowering CRP levels, reducing vascular inflammatory responses, and improving endothelial cell function, with positive effects on preventing and reducing cardiovascular clinical endpoints [31]. This study also showed that statin use was higher in the group with normal CRP than in the group with elevated CRP, and although not statistically significant, statin use reduced the risk of hypertension (OR = 0.49,95%CI: 0.25–0.94, *p* < 0.05) and was protective against hypertension. Earlier studies have suggested an association between alcohol consumption and CRP, but the form of the association is uncertain [32]. Our study found a higher proportion of alcohol consumption in the group with elevated CRP (*P* < 0.05), which is similar to the findings of a recent cohort study of Chinese men aged 50 years and older [33], which showed that compared with never and occasional drinkers (< 1 drink/week), daily (OR = 1.38, 95% CI: 1.24–2.65) and excessive drinkers (weekly ethanol intake ≥ 210 g, OR = 1.57, 95% CI: 1.22–2.02) had a higher risk of elevated CRP and a higher risk of cardiovascular disease, and therefore patients are advised to drink less or even abstain from alcohol.

As a retrospective study with elderly patients, the data were obtained from the electronic medical records of patients attending our hospital, based on the patients' admission, the collection of general data may lack accuracy, thus affecting the interpretation of the results. Furthermore, we included elderly patients, and because co-morbidity is common in elderly patients, it was not possible to set up a healthy population as a control group to assess the relationship between CRP and hypertension. Finally. our study, as a cross-sectional study, was unable to assess the relationship between CRP concentration and the severity of hypertension, with a small sample size, and further prospective analysis is needed in the future.

## Conclusions

The present study showed that elevated CRP was positively associated with hypertension in the elderly, and in addition to CRP, BMI and diabetes mellitus were also shown to be risk factors for hypertension, and statin use may reduce the inflammatory response to some extent and have a protective effect on hypertension. In addition, alcohol consumption, LDL-C, coronary artery disease, arthritis, and steroid use are also associated with elevated CRP. The inflammatory response is involved in the development of hypertension, and early screening for CRP in the elderly population and early initiation of treatment may help prevent further development of the inflammatory response, and increasing the sample size will facilitate further research on whether CRP can be used to manage hypertension and other related diseases in the elderly.

**Table 1:** Comparison of baseline information between the group with elevated CRP and the group with normal CRP

*Variable*	*Elevated CRP (n* = *120)*	*Normal CRP (n* = *76)*	*P-value*
Age (years)	72.5(68.0,78.0)	73.0(68.3,77.0)	NS
Gender			
Male (n, %)	64.0(53.3)	45.0(59.2)	NS
Female (n, %)	56.0(46.7)	31.0(40.8)	
BMI(kg/m2)	24.6 ± 4.2	24.2 ± 4.1	NS
Cigarettes (n, %)	16.0(13.3)	13.0(17.1)	NS
Alcohol (n, %)	31.0(25.8)	10.0(13.2)	< 0.05^*^
SBP (mmHg)	142.9 ± 20.5	136.7 ± 19.2	< 0.05^*^
DBP (mmHg)	78.7 ± 12.3	75.3 ± 9.4	< 0.05^*^
Laboratory Metrics			
TC (mmol/L)	4.0(3.5,5.3)	3.9(3.2,4.8)	NS
TG (mmol/L)	1.1(0.8,1.5)	1.2(0.7,1.6)	NS
LDL-C(mmol/L)	2.8(2.3,3.5)	2.5(2.0,3.2)	< 0.05^*^
HDL-C(mmol/L)	1.1(0.9,1.3)	1.1(0.9,1.5)	NS
WBC (10^9^/L)	7.0(5.2,8.9)	6.6(5.3,8.1)	NS

Data are presented as frequency (percentage) for categorical variables and as mean ± standard deviation or median(interquartile range) for continuous variables

*NS* not significant, *BMI* body mass index, *SBP* Systolic blood pressure, *DBP* Diastolic blood pressure, *TC* total cholesterol, *TG* triglyceride, *LDL-C* low-density lipoprotein cholesterol, *HDL-C* high-density lipoprotein cholesterol, *WBC* White blood cells

*indicates statistically significant difference

**Table 2:** Comparison of medication use between patients in the group with elevated CRP and those in the group with normal CRP (n, %)

*Variable*	*Elevated CRP (n* = *120)*	*Normal CRP (n* = *76)*	*P-value*
Aspirin (n, %)	32.0(26.7)	16.0(21.1)	0.373
Statin (n, %)	39.0(32.5)	26.0(34.2)	0.804
NSAIDS (n, %)	55.0(45.8)	29.0(38.2)	0.290
Steroid (n, %)	54.0(45.0)	23.0(30.3)	0.040^*^
Immunosuppressive agents (n, %)	40.0(33.3)	17.0(22.4)	0.100

*NSAIDs*, Non-steroidal anti-inflammatory drugs

^*^indicates statistically significant difference

**Table 3:** Comparison of co-morbidities between the group with elevated CRP and the group with normal CRP (n, %)

*Variable*	*Elevated CRP (n* = *120)*	*Normal CRP (n* = *76)*	*P-value*
Hypertension (n, %)	85.0(70.8)	39.0(51.3)	0.006^*^
Diabetes mellitus (n, %)	38.0(31.7)	18.0(23.7)	0.228
Hyperlipidemia (n, %)	31.0(25.8)	18.0(23.7)	0.735
Coronary heart disease (n, %)	54.0(45.0)	22.0(28.9)	0.025^*^
Cerebrovascular disease (n, %)	41.0(34.2)	20.0(26.3)	0.247
Carotid artery stenosis (n, %)	6.0(5.0)	2.0(2.6)	0.656
Peripheral arterial disease (n, %)	35.0(29.2)	23.0(30.3)	0.870
Hypothyroidism (n, %)	8.0(6.7)	7.0(9.2)	0.514
Congestive heart failure (n, %)	13.0(10.8)	13.0(17.1)	0.207
Arthritis (n, %)	31.0(25.8)	10.0(13.2)	0.034^*^
Other rheumatologic disorders (n, %)	29.0(24.2)	15.0(19.7)	0.469
Psychosomatic diseases (n, %)	4.0(3.3)	4.0(5.3)	0.768
COPD (n, %)	14.0(11.7)	10.0(13.2)	0.756
Asthma (n, %)	6.0(5.0)	1.0(1.3)	0.337
Liver disease (n, %)	6.0(5.0)	9.0(11.8)	0.079

*COPD* Chronic obstructive pulmonary disease

^*^indicates statistically significant difference

**Table 4:** Multi-factor logistic regression analysis of factors associated with CRP and hypertension

*Variable*			
Age (years)		1.02(0.97–1.07)	0.568
BMI(kg/m2)		1.10(1.01–1.19)	0.030^*^
Gender	Female		
	Male	0.69(0.36–1.31)	0.256
CRP	Normal		
	High	2.13(1.14–3.99)	0.019^*^
Diabetes mellitus	No		
	Yes	2.68(1.24–5.79)	0.012^*^
Asthma	No		
	Yes	2.60(0.28–24.55)	0.404
COPD	No		
	Yes	0.89(0.33–2.40)	0.819
Statin	No		
	Yes	0.49(0.25–0.94)	0.033^*^

*BMI* body mass index, *CRP* c-reactive protein, *COPD* Chronic obstructive pulmonary disease, *OR* odds ration

^*^indicates statistically significant difference

## Data Availability

The datasets used and/or analysed during the current study available from the corresponding author on reasonable request.
